# Letter from the Editor in Chief

**DOI:** 10.19102/icrm.2025.16038

**Published:** 2025-03-15

**Authors:** Devi Nair


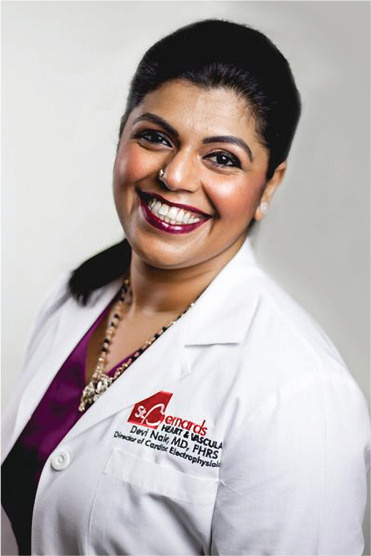


Dear Colleagues,

As we enter the spring of 2025, I am pleased to present this March issue of *The Journal of Innovations in Cardiac Rhythm Management*. This edition is uniquely rich, featuring breakthroughs in arrhythmia mechanisms, innovative therapeutic strategies, and a special focus on the next generation of electrophysiologists.

This month’s issue coincides with our reflections on the Western Atrial Fibrillation Symposium, which featured a day of live and recorded cases for the first time, allowing for an immersive discussion on procedural best practices and real-world challenges. The scientific agenda was invigorating, with deep dives into the mechanisms of atrial fibrillation (AF) and critical debates about the future direction of AF treatment.

## The Next Generation of Electrophysiologists: Recognizing Fellows’ Contributions

This month, we highlight an exciting contribution to the journal: a Letter from the Directors of the 2024 Electrophysiology Fellows Summit, Drs. William Sauer and Wendy Tzou, recognizing the outstanding case reports and research efforts of early-career electrophysiologists. The Electrophysiology Fellows Summit serves as a premier educational forum, bringing together established experts and emerging leaders in the field to share novel insights, innovative case studies, and cutting-edge procedural techniques.

Three case report finalists were selected to present at this year’s EP Fellows Summit Case Competition, covering a broad spectrum of clinical challenges:

- ***Non-pulmonary vein triggers masked by general anesthesia.*** This case highlighted the challenges in identifying hidden AF mechanisms and the strategies to improve procedural success.
- ***Syncope due to coronary vasospasm and atrioventricular block.*** This is a fascinating case illustrating the interplay between coronary pathology and conduction system disturbances.
- ***Pulsed field ablation combined with radiofrequency for ventricular tachycardia.*** This case introduces an innovative approach demonstrating the potential synergy of different energy modalities to improve ablation outcomes.

Congratulations to Dr. James Mannion for his winning case and to Drs. Sang Lee and Maxwell Coll for their impactful presentations. These fellows are shaping the future of electrophysiology, and we look forward to their continued contributions to the field.

## Featured Research in This Issue

This month’s journal also includes original research and case reports exploring some of the most thought-provoking and clinically relevant topics in electrophysiology:

- ***“The Rhythms of the Moon: Can Lunar Phases Influence Cardiac Arrhythmias?.”*** Dr. Athanasios Ziakos and colleagues provide a data-driven analysis debunking common patient-held beliefs about the influence of the lunar cycle on arrhythmia occurrence. With an extensive dataset spanning nearly a decade, their findings reaffirm that cardiac arrhythmias are independent of moon phases or the Earth–Moon distance.
- ***“Immune Checkpoint Inhibitor–induced Myocarditis Leading to Complete Heart Block.”*** Dr. Viraj Panchal and team present a compelling case of pembrolizumab-induced myocarditis, a growing concern in patients receiving cancer immunotherapy. This case report underscores the importance of early detection, aggressive management, and a multidisciplinary approach to immune-mediated cardiac toxicity.

## Western AF Symposium 2025: Where Do We Go from Here?

The Western AF Symposium continues to be an essential platform for advancing our understanding of AF mechanisms and refining treatment strategies. This year, a particular emphasis was placed on challenging conventional thinking and questioning long-held assumptions about AF progression and ablation outcomes.

Key themes and questions included:

- ***Live and recorded case sessions.*** For the first time, a full day was dedicated to real-world case discussions, providing attendees with a hands-on, case-based approach to learning. These cases underscored both the complexities and successes of modern AF treatment.
- ***Understanding AF mechanisms: have we been approaching this the right way?*** What truly drives persistent AF? Should we rethink our lesion sets and mapping strategies? Finally, are we correctly identifying the arrhythmogenic substrates, or are we missing critical elements of AF pathophysiology?
- ***The future of AF treatment: do we need a new path forward?*** What is the role of hybrid ablation strategies—should thermal and pulsed field energy be combined, or are we introducing new risks? How can we better personalize therapy to improve long-term AF freedom rates? Finally, when do we stop ablating? Are we over- or under-delivering energy, and how can we establish more standardized procedural endpoints?

These fundamental questions will shape the next decade of AF research and treatment, and, as a field, we must remain open to re-evaluating our current paradigms.

## Looking Ahead

This issue reflects the dynamism of the electrophysiology community—from experienced experts pushing the boundaries of innovation to early-career fellows bringing fresh perspectives and exciting case reports. As the field advances, our collective responsibility is to balance innovation with rigor, ensuring that new technologies and treatment strategies are guided by evidence and optimized for patient safety and effectiveness.

I extend my deepest gratitude to all contributors, reviewers, and readers who continue to drive progress in cardiac rhythm management. As we look forward, let us remain curious, collaborative, and committed to refining our approaches to AF management and beyond.

Warm regards,


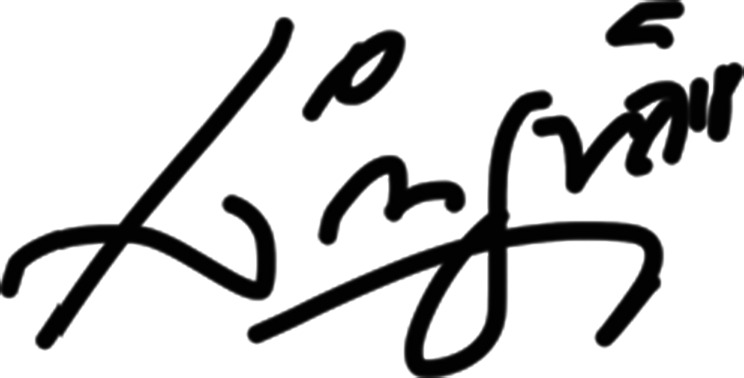


Dr. Devi Nair, md, facc, fhrs

Editor-in-Chief

*The Journal of Innovations in Cardiac Rhythm Management*

Director of the Cardiac Electrophysiology & Research,

St. Bernard’s Heart & Vascular Center, Jonesboro, AR, USA

White River Medical Center, Batesville, AR, USA

President/CEO, Arrhythmia Research Group

Clinical Adjunct Professor, University of Arkansas for Medical Sciences

Governor, Arkansas Chapter of American College of Cardiology

drdgnair@gmail.com

